# Diffuse Large B-Cell Lymphoma: Presentation at an Early Age

**DOI:** 10.7759/cureus.74208

**Published:** 2024-11-22

**Authors:** Sandra Fernandes

**Affiliations:** 1 Family Medicine, ARS Médica Family Health Unit, Local Health Unit Loures-Odivelas, Lisbon, PRT

**Keywords:** family/general practice, hemathology, large b-cell lymphoma, non-hodgkin lymphoma (nhl), young people

## Abstract

Diffuse large B-cell lymphoma (DLBCL) is a clinical entity that constitutes a large part of non-Hodgkin lymphoma (NHL). It usually presents as a rapidly growing mass or with enlarged lymph nodes in a nodal or extranodal location. It most frequently affects Caucasians, with a male preponderance and an average age of onset of around 60 years. The patient, in this case, was a young adult, with irrelevant personal and family history, who presented with sudden onset abdominal pain and asthenia. An objective examination of the abdomen demonstrated a hard mass in the region of the lower quadrants that caused pain when touched. Given the patient's non-specific symptoms and age, it would be more likely to think about less complex diagnoses; however, a thorough observation of the patient led to the possibility of a less likely diagnosis. This case highlights the importance of a detailed anamnesis and an attentive and targeted objective examination in order to guarantee a correct diagnosis.

## Introduction

According to the World Health Organization (WHO), diffuse large B-cell lymphoma (DLBCL) is characterized by a diffuse proliferation of lymphoid B cells [1]. This clinical entity represents approximately 30% of all cases of non-Hodgkin's lymphoma (NHL) [2], with an annual estimate of 150,000 new cases worldwide. It is more common in males and the average age at diagnosis is around 60-65 years, with 30% of patients over 75 years of age [3].

The etiology of this clinical entity is multifactorial and risk factors include genetic characteristics, immunological dysregulation, and exposure to viral, environmental, or occupational agents. The clinical presentation is usually a rapidly growing symptomatic mass, usually with enlarged lymph nodes in the neck or abdomen [4]. Symptoms B (fever, weight loss, and night sweats) are observed in about 30% of cases [5,6].

The diagnosis is usually made through an excisional biopsy of the lymph node. The pathological diagnosis is based on morphology and immunophenotyping. The architecture of the lymph node is generally obliterated by layers of large, atypical lymphocytes with prominent nucleoli and basophilic cytoplasm, a diffuse growth pattern, and a high proliferative index. Tumor cells generally express B cell antigens (CD19, CD20, CD22. CD79a); however, there is no cytogenetic alteration that is pathognomonic of the diagnosis [1].

The prognosis considers tumor histopathology and the International Prognostic Index (IPI), which encompasses the patient's age, serum lactate dehydrogenase concentration, Eastern Cooperative Oncology Group (ECOG) performance status, Ann Arbor classification, and number of sites with extranodal pathology [7]. Approximately two-thirds of patients diagnosed with DLBCL can be cured with chemotherapy consisting of Rituximab [8]. Recognizing this clinical entity early is important to raise the survival rate in patients with this diagnosis. 

## Case presentation

An 18-year-old male patient presented to the hospital emergency department with moderate, generalized, and sudden onset abdominal pain. He classified the pain as 6/10 on the numerical pain scale. He denied irradiation and relieving or worsening factors and highlighted that a month before, he had a similar episode, with spontaneous resolution. He denied fever, gastrointestinal or genitourinary changes, weight loss, night sweats, or anorexia. He stayed with his mother and siblings in good hygiene conditions. Past medical history included an intra-abdominal abscess one year ago, for which he underwent conservative treatment. There was no relevant family history, habitual medication, use of psychoactive substances, risky sexual contacts, or drug allergies.

The patient was conscious and oriented. Upon physical examination, he was seen to be in good general condition. Skin and mucous membranes were flushed and hydrated, cardiopulmonary auscultation was normal and hemodynamically stable. The abdomen was stiff, depressible, and slightly painful on palpation in the lower quadrant region, with greater expression at the hypogastric level, where a mass was palped.

An analytical evaluation was requested, which demonstrated neutrophilia, with changes in liver parameters with an increase in aspartate aminotransferase, alanine aminotransferase, gamma-glutamyl transferase, and lactic dehydrogenase. There was also an increase in the level of C-reactive protein. Urine II sample (summary urine analysis) had no changes. The values ​​of laboratory tests are presented in Table 1

**Table 1:** Laboratory values at presentation at the emergency department

Tests	Patient values	Reference values
Erythrocytes	5.27 10E6/mm³	4.4-5.9 10E6/mm³
Hemoglobin	14.3 g/L	13-17 g/L
Hematocrit	42.9 %	40-50%
Mean corpuscular volume	81.4 fL	78-96 fL
Leukocytes	9.51 10E6/mm³	4.5-11 10E6/mm³
Neutrophils	80.6 %	40-75 %
Platelets	351 10E6/mm³	150-450 10E6/mm³
Creatinine	1.02 mg/dL	0.7-1.10 mg/dL
Prothrombin time	13.9 sec	9.4-12.5 sec
International normalized ratio	1.18	0.8-1.20
Aspartate aminotransferase	42 U/L	<32 U/L
Alanine aminotransferase	36 U/L	<18 U/L
Gamma-glutamyl transferase	19 U/L	4-16 U/L
Lactic dehydrogenase	266 U/L	0-240 U/L
C-reactive protein	26.9 mg/L	< 5 mg/L

**Figure 1:**
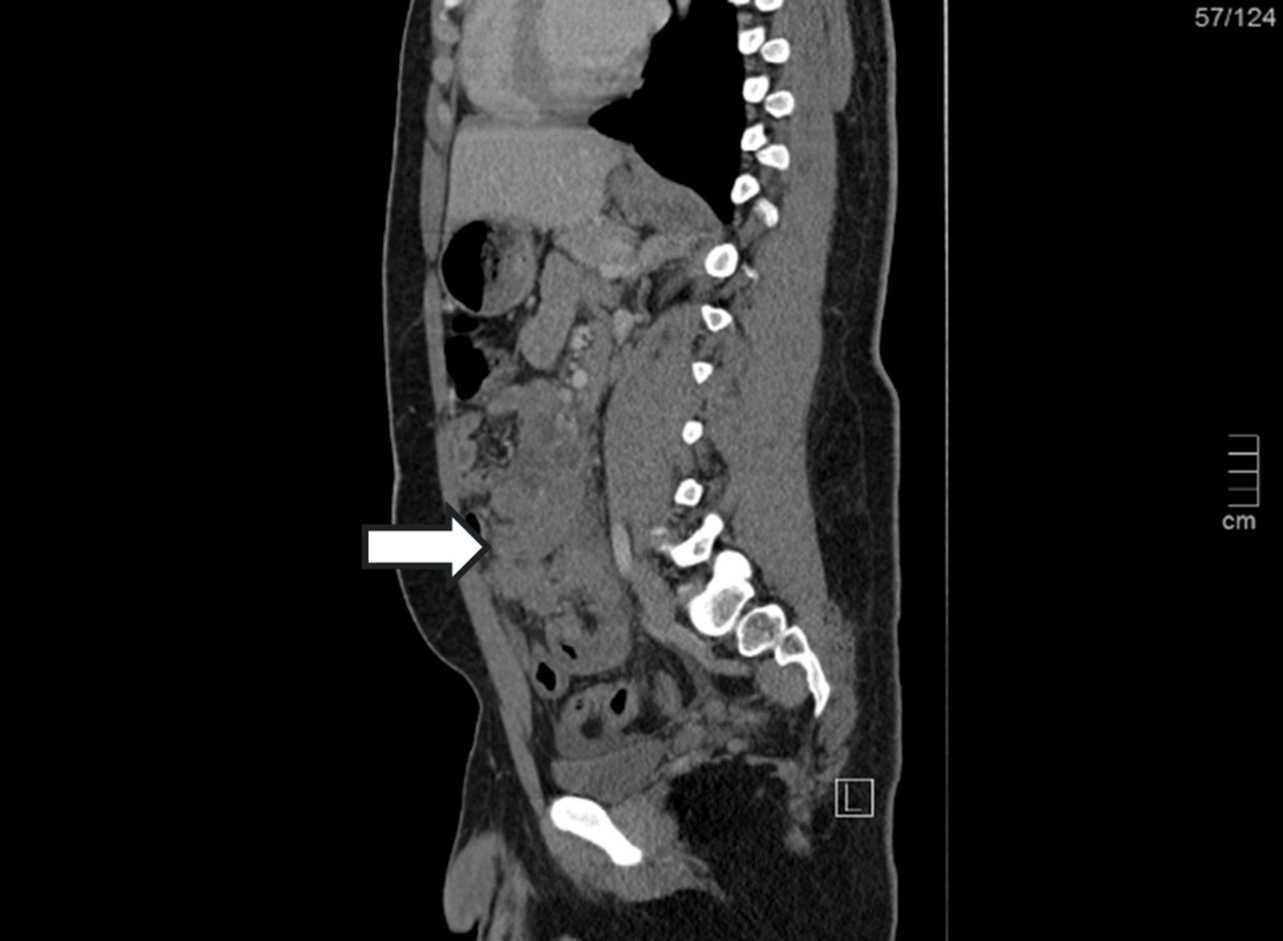
Abdominopelvic CT showing adenopathic conglomerate (side view)

**Figure 2:**
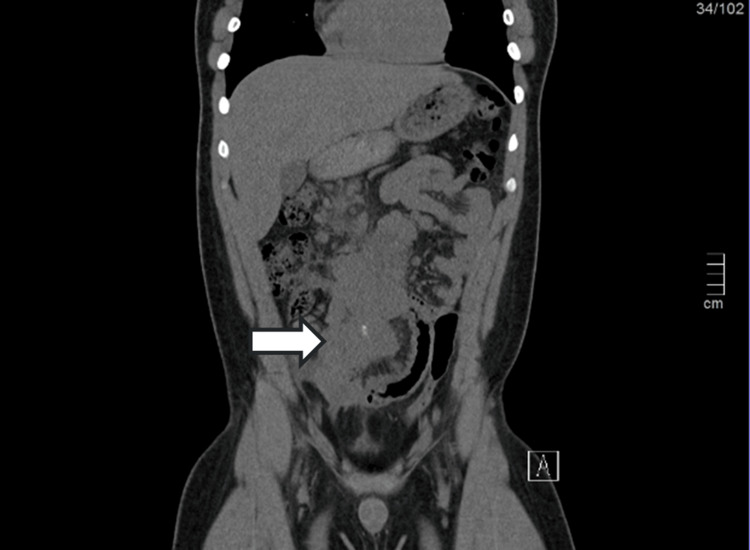
Abdominopelvic CT showing adenopathic conglomerate (front view)

After the analytical evaluation, an abdominopelvic CT was done which revealed an adenopathic conglomerate measuring 9x8 cm, involving the root of the mesentery and with encasement of the superior mesenteric artery and marked parietal thickening of the small intestine loop in the pelvic excavation, approximately 7 cm in longitudinal axis. No fluid collections were observed in the abdominal cavity or free fluid intraperitoneally (Figure 1 and Figure 2).

To clarify the adenopathic conglomerate diagnosis, the patient was admitted for a biopsy which revealed a proliferation of poorly differentiated neoplastic cells; an immunohistochemical study revealed positivity for CD20, Vimentin, BCL2, BCL6, and focal CD5, confirming the existence of a lymphoma, B, CD20 +, diffuse large cells, with proliferative index Ki67 of 80%. A CT of the chest and neck was performed for staging, which revealed no acute changes, and the patient was referred to another hospital, with a Hematology unit for discussion and treatment implementation.

## Discussion

DLBCL is an aggressive B-cell neoplasm, constituting most cases of NHL [8]. It typically occurs in male individuals, with an average age of around 60-65 years, and is in most cases paucisymptomatic. In the present case, the patient was young, paucisymptomatic, and without important personal or family history. The patient's age could be a confounding factor, as in this case, making us refer to other more likely pathologies.

It is important that clinicians always have a critical look at what they observe, regardless of the biases found. In view of the case presented, more common diagnostic hypotheses could have been listed for the patient's complaints and considering his age group (notably gastroenteritis), which presents a benign course. The abdominal mass found during the targeted objective examination, which was not compatible with the clinical entities most likely to be the cause of the symptoms presented by the patient, led to a routine analytical evaluation, which was the decisive step in requesting an imaging exam that made the definitive diagnosis more certain. The biopsy led to the final diagnosis and respective therapeutic guidance. If the final diagnosis were inferred, based on the patient's age, without questioning the findings of the objective examination, this would be erroneous and would have led to a delay in diagnosing DLBCL and thus delay in instituting therapy, which would ultimately have a negative impact on the patient's life prognosis [1,8].

Understanding the impact of this disease on a young adult, not only on a physical level but also on a mental and emotional level, is also part of the doctor's work in an emergency context, and he or she can refer the patient to a Mental Health consultation. It is also important to develop techniques for communicating bad news, to facilitate patient understanding and increase therapeutic adherence. Family doctors can make a difference in these cases, since being a generalist specialty, they must always take into account the clinical history and the directed physical examination. We must always consider the typical characteristics of the different clinical entities, while objectively looking at the clinical findings found, which can guide us towards alternative diagnoses to those initially considered.

## Conclusions

DLBCL is an aggressive hematological pathology, which often begins at older ages. When we come across “suspicious” findings on objective examination, we must have a high degree of suspicion and guide our actions more broadly, researching even pathologies that are less common in the patient’s age group. Considering the aggressiveness of this clinical entity and its prognosis, treatment must be instituted peremptorily. Family doctors, who have a generalist specialty, have a privileged clinic vision to recognize clinical findings that are not consistent with the most common characteristics of the various pathologies.
